# Limited Value of HBV‐RNA for Relapse Prediction After Nucleos(t)ide Analogue Withdrawal in HBeAg‐negative Hepatitis B Patients

**DOI:** 10.1111/jvh.14026

**Published:** 2024-10-19

**Authors:** Valerie Ohlendorf, Maximilian Wübbolding, Christoph Höner zu Siederdissen, Birgit Bremer, Katja Deterding, Heiner Wedemeyer, Markus Cornberg, Benjamin Maasoumy

**Affiliations:** ^1^ Department of Gastroenterology, Hepatology, Infectious Diseases and Endocrinology Hannover Medical School Hannover Germany; ^2^ Centre for Individualised Infection Medicine (CiiM) A Joint Venture of Helmholtz Centre for Infection Research and Hannover Medical School Hannover Germany; ^3^ German Center for Infection Research (DZIF) Partner‐Site Hannover‐Braunschweig Hannover Germany; ^4^ Cluster of Excellence RESIST (EXC 2155) Hannover Medical School Hannover Germany; ^5^ TWINCORE, Centre for Experimental and Clinical Infection Research A Joint Venture Between the Hanover Medical School and the Helmholtz Centre for Infection Research Braunschweig Germany

**Keywords:** chronic hepatitis B, Nucleos(t)ide analogue, pregenomic RNA, relapse, treatment cessation

## Abstract

International guidelines suggest cessation of nucleos(t)ide analogues (NA) independent of HBsAg loss in HBeAg‐negative patients after 2–3 years of viral suppression. Detectable HBV‐RNA levels at the time of NA cessation were linked to a better prediction of relapse after NA withdrawal in small cohorts of HBeAg‐negative patients. This study proves the impact of HBV‐RNA levels in the prediction of relapse in a large cohort of HBeAg‐negative patients, mainly infected with genotype B or C. Serum levels of HBV‐RNA, HBsAg, anti‐HBc and HBcrAg were determined before NA withdrawal in 154 HBeAg‐negative patients, participating either in a therapeutic vaccination trial (NCT02249988) or in an observational register trial (NCT03643172). Importantly, vaccination showed no impact on relapse. Endpoints of the study were virological relapse (HBV‐DNA > 2000 IU/mL) or biochemical relapse (attendant ALT levels ≥ 2 × ULN) 24 weeks after NA cessation. Virological relapse occurred in 54.5% of patients (*N* = 84/154), including eight patients (10%) developing an ALT flare. Baseline HBV‐RNA level did not differ significantly between relapsers and off‐treatment responders (*p* = 0.92). No significant difference occurred in proportions of detectable HBV‐RNA levels between off‐treatment responders (*N* = 27/70; 38.6%) and relapsers (*N* = 31/84; 36.9%) (*p* = 0.99). Combining predefined HBsAg cut‐offs (100 IU/mL, *p* = 0.0013), anti‐HBc cut‐offs (325 IU/mL, *p* = 0.0117) or HBcrAg cut‐offs (2 log U/mL, *p* = 0.66) with undetectable HBV‐RNA (HBsAg, *p* = 0.0057; anti‐HBc, *p* = 0.085; HBcrAg, *p* = 0.60) did not improve relapse prediction. The value of HBV‐RNA levels at timepoint of NA cessation for the prediction of relapse is limited in HBeAg‐negative patients.

**Trial Registration:** ABX 203‐002: NCT02249988; Terminator 2: NCT03643172

**Abbreviations**
ALT: alanine transaminase
anti‐HBc: antibody against hepatitis B core antigen
BL: baseline
BR: biochemical relapse
cccDNA: covalently closed circular DNA
cHBV: chronic hepatitis B virus infection
EoS: end of study
EoT: end of treatment
ETV: entecavir
HBcrAg: hepatitis B core‐related antigen
HBeAg: hepatitis B virus e antigen
HBsAg: HBs antigen
HBV: hepatitis B virus
HCC: hepatocellular carcinoma
HCV: hepatitis C virus
HDV: hepatitis D virus
HIV: human immunodeficiency virus
NA: nucleos(t)id analogue
pgRNA: pregenomic hepatitis B virus RNA
TDF: tenofovir
VR: virological relapse

## 1 Introduction

Chronic hepatitis B virus infection (cHBV) with its long‐term complications, comprises the development of liver cirrhosis or hepatocellular carcinoma (HCC), still represents a serious public health problem worldwide [1].

Nucleos(t)ide analogues (NA) lead to virological and biochemical response in most patients and thereby reduce the risk of liver fibrosis and HCC development [2, 3, 4]. NA inhibits the reverse transcriptase activity of the HBV polymerase, but does not sufficiently suppress de novo synthesis of the nuclear covalently closed circular DNA (cccDNA), resulting in a persisting HBV‐RNA and HBV protein synthesis [5]. Accordingly, virological relapse (with or without an attended hepatitis) after NA withdrawal is common [6]. Especially for hepatitis B e antigen (HBeAg)‐negative patients, where basal core promotor mutations or precore mutations are commonly detected, high relapse rates up to 57% one year after therapy cessation are recognised [7, 8].

International guidelines consistently suggest therapy continuation until HBsAg loss in HBeAg‐negative patients [9]. However, a functional cure (defined as HBV‐DNA and hepatitis B surface antigen (HBsAg) seroclearance with or without HBsAg seroconversion) is only rarely recognised under NA treatment and NA cessation can lead to severe flares with decompensation of the liver function [8, 10, 11]. Although in Asian populations an end‐of‐treatment (EoT) HBsAg level < 100 IU/mL was described to be associated with low rates of relapse, even this cut‐off is attained only in a minority of patients [12]. Therefore, long‐term treatment is necessary for most of the cHBV‐infected patients. Considering the significant financial burden and the burden of life‐long medication, a surrogate marker that helps to estimate the outcome of NA discontinuation before HBsAg seroclearance was studied intensively in the last decade [4, 13, 14].

HBV‐RNA has been shown to correlate with the intrahepatic cccDNA transcription activity and was, therefore, postulated to be a potential biomarker in the prediction of relapse after NA withdrawal [15, 16]. The majority of studies investigating HBeAg positive or mixed cohorts of HBeAg positive and negative patients reported evidence that EoT HBV‐RNA level, alone or in combination with HBsAg or hepatitis B core‐related antigen (HBcrAg), is a useful biomarker in the prediction of virological and biochemical relapse after NA cessation [17, 18, 19]. But so far, only four studies with small cohorts of HBeAg‐negative patients were performed in this context, reporting discrepant results concerning the association of HBV‐RNA and the risk of biochemical or virological relapses respectively [20, 21, 22, 23].

This study aimed to contribute to the so far published results by investigating the association of serum HBV‐RNA levels alone or in combination with HBsAg, HBcrAg or HBc antibody (anti‐HBc) levels with relapse after NA withdrawal in a large cohort of exclusively HBeAg‐negative patients, mainly infected with HBV genotype B and C.

## 2 Methods

The analysed HBeAg‐negative cohort was selected from patients participating either in the ABX 203‐002 study (NCT02249988) or in an observational register trial (Terminator 2, NCT03643172). Both studies were conducted in accordance with the declarations of Helsinki and Istanbul. The presented analysis of biomarker and anonymous patient data was additionally approved by the local ethics committee of Hannover Medical School (10556_BO_K_2022). Likewise, the observational register trial Terminator 2 was approved by the local ethics committee of Hannover Medical School (7982_BO_K_2018). All included subjects gave written informed consent for the respective study participation.

### 2.1 Study Cohort

The ABX 203‐002 study (a phase 2b‐3, open‐label, randomised, multicentre study from the Asia Pacific region) investigated the capacity of the vaccine ABX 203‐002 for a sustained control of cHBV infection in HBeAg‐negative patients after cessation of antiviral treatment. Patients enrolled in the study received NA for at least 2 years, in line with the Asian‐Pacific stopping criteria [24]. Before NA cessation, a proportion of the participants additionally received the vaccine ABX203 over 20 weeks. Importantly, vaccination did not show any effect on the risk for virological/biochemical relapse after stop of NA treatment (*p* = 0.71). Inclusion and exclusion criteria of the ABX 203‐002 study were described previously by Höner zu Siederdissen et al. [25].

Terminator 2 is an observational register trial that aimed to investigate and confirm the feasibility and safety of NA cessation in European HBeAg‐negative cHBV patients. Patients enrolled in the study must have a detectable HBsAg and must be on an ongoing therapy with entecavir (ETV) or tenofovir (TDF) with a stable virological suppression (HBV‐DNA < 20 IU/mL) for at least 3 years prior to NA cessation, according to the EASL clinical practice guidelines [4]. Exclusion criteria comprised signs of advanced liver fibrosis or cirrhosis (liver stiffness > 10.0 kPa measured by FibroScan [26] or evidence of bleeding from oesophageal varices or other conditions consistent with decompensated liver disease), evidence of a medical condition associated with chronic liver disease other than HBV, evidence of HDV‐, HIV‐ or HCV‐coinfection, history of organ transplantation or history of immunomodulatory treatment 12 month prior to NA cessation or hepatocellular carcinoma.

### 2.2 Study Design

Serum levels of HBV‐RNA, anti‐HBc, HBsAg and HBcrAg of included patients were determined at baseline (BL), defined as 24 weeks before NA cessation (ABX 203‐002, time point of the first vaccination) or 12 weeks before NA cessation (observational register trial Terminator 2) respectively. Furthermore, HBV‐DNA as well as ALT/AST level were determined at BL and were additionally analysed every 2–4 weeks after treatment cessation until 24 weeks after NA withdrawal (end of study, EoS).

The endpoint of the study was relapsing 24 weeks after treatment cessation and subdivided into virological relapse (VR), defined as HBV‐DNA > 2000 IU/mL with ALT levels ≤ 2× ULN, biochemical relapse (BR), defined as HBV‐DNA > 2000 IU/mL and ALT levels ≥ 2× ULN and flare, defined as HBV‐DNA > 2000 IU/mL and ALT levels > 5× ULN respectively. Importantly, NA treatment was reinitiated in patients with an increase of HBV‐DNA ≥ 2000 IU/mL and/or ALT/AST ≥ 5× ULN.

An overview of the structure of the presented study is given in Figure S1.

### 2.3 Laboratory Testing

Baseline serum level of HBV‐RNA was obtained using the Cobas HBV‐RNA investigational PCR assay and the Cobas 6800/8800 system, following the manufacturer's protocol (Roche Molecular Systems, Pleasanton, California, USA). Serum level of anti‐HBc and HBcrAg was measured using the Lumipulse G HBcAb‐N Immunoreaction assay (Fujirebio Europe, Belgium). The HBV genotype was identified for patients with detectable HBV‐DNA ≥ 500 IU/mL as described before [27].

Quantification of HBV‐DNA from samples obtained from the ABX 203‐002 study was performed using the Roche Cobas AmpliPrep/Cobas TaqMan HBV Test (version 2.0; Roche Diagnostics, Basel, Switzerland). For the quantification of HBV‐DNA and HBsAg level in samples from patients participating in the Terminator 2 trial, the Aptima HBV Quant Assay running on the fully automated Panther system (Hologic, Marlborough, Massachusetts, USA) and the ARCHITECT HBsAg assay (Abbott, North Chicago, Illinois, USA) were used respectively.

### 2.4 Statistical Analysis

Statistical analyses were performed using the software package SPSS Statistics v.25.0 (IBM Corp., Armonk, NY) and the software GraphPad Prism version 9.1.2 (GraphPad Software, San Diego, California USA). A *p* value of < 0.05 was considered to be statistically significant.

Since the lower limit of detection of the HBcrAg assay is 2 log U/mL, HBcrAg levels lower than 2 log U/mL were calculated as 2 log U/mL for statistical analysis as previously described [28, 29]. The lower limit for the quantification of HBV‐RNA was 10 copies/mL. Detectable but not quantifiable HBV‐RNA levels (< 10 copies/mL) were set to 10 copies/mL for statistical analysis.

The Mann–Whitney *U* test was used to compare baseline HBV‐RNA levels between relapsers and off‐treatment responders. Significance of categorical variables was analysed using Fisher's exact test. To analyse longitudinal data, the Kaplan–Meier analysis was used with the log‐rank test for the consideration of significance. Correlation between HBV‐RNA levels and anti‐HBc, HBsAg and HBcrAg levels was tested with Spearman correlation.

Proportions of patients with relapse were determined and categorised by predefined HBsAg, HBV‐RNA and HBcrAg cut‐offs, analogue to previously performed studies [20, 21]. The optimal anti‐HBc and HBV‐RNA cut‐off of the investigated cohort for the prediction of relapse was determined by the Youden index. The diagnostic value of the anti‐HBc level was assessed using the area under the receiver operating characteristic curve (AUROC), determined by the Wilson/Brown method.

Binary logistic regression analyses were performed to identify predictors of relapse, using common criteria in clinical trials (confidence interval [CI]: 95%; statistical level of significance: 5%). Binary logistic regression analyses were adjusted for age, therapy duration, HBV genotype B, C, D as well as HBV‐RNA, anti‐HBc, HBsAg and HBcrAg levels to BL.

## 3 Results

### 3.1 Baseline Characteristics of the Study Population

A total of 154 HBeAg‐negative cHBV patients were included in this study. HBV‐RNA was detectable in 37.7% (*N* = 58/154) of patients at BL. Among participants with detectable HBV‐RNA at BL, the median HBV‐RNA level was 10 copies/mL (10–1.52 × 10^3^ copies/mL). Median baseline HBV biomarker levels of the overall cohort were HBV‐RNA 0 copies/mL (0–1.52 × 10^3^ copies/mL), anti‐HBc 410 IU/mL (11.2–7.9 × 10^3^ IU/mL), HBsAg 949.5 IU/mL (2–4.0 × 10^4^ IU/mL) and HBcrAg 3.1 log U/mL (2–5.5 log U/mL) respectively. The most abundant HBV genotypes were genotypes B and C (22.1% and 28.6%) respectively. Further baseline characteristics of the study population are presented in Table 1, and baseline parameters of the subpopulations according to the respective study participation are presented in Table S1.

**TABLE 1:** Baseline characteristics of the study population.

Parameter		*N* (Total cohort = 154)
Age (in years)		52.5 (20–66)
Male sex		115 (75%)
Nucleos(t)id analogue	Entecavir	94 (61%)
	Tenofovir	60 (39%)
Therapy duration (in months)		61 (30–257)
HBeAg negativity		154 (100%)
HBV‐DNA (< 20 IU/mL)		154 (100%)
ALT (U/mL)		21.5 (6–71)
Transient elastography (kPa)		5.2 (2.7–9.9)
Platelets (thousand/μl)	—	196 (81–348)
Bilirubin (mmol/l)	—	9 (3–36)
Anti‐HBc level (IU/mL)	—	410 (11.2–7.9 × 10^3^)
HBsAg level (IU/mL)	—	949.5 (2–4.0 × 10^4^)
HBcrAg level (log U/mL)	—	3.1 (2–5.5)
HBV‐RNA (copies/mL)		10 (0–2.4 × 10^3^)
Genotype		
A	—	3 (1.9%)
B	—	34 (22.1%)
C	—	44 (28.6%)
D	—	11 (7.1%)
E	—	1 (0.6%)
Unknown		61 (39.6%)

*Note:* Continuous variables are expressed in median (min–max).

### 3.2 HBV‐RNA Level and the Prediction of Relapse

The median time to relapse of the overall cohort was 8 weeks (2–24 weeks) after therapy cessation. Of note, included participants of the observational register trial Terminator 2 developed no further relapse between weeks 24 and 130 after EoT. VR (HBV‐DNA > 2000 IU/mL) occurred in 49.4% of patients (*N* = 76/154) and eight patients (5.2%) developed a BR (HBV‐DNA > 2000 IU/mL + ALT levels ≥ 2× ULN), including five patients (3.25%) with a flare (HBV‐DNA > 2000 IU/mL + ALT levels > 5× ULN). Eight patients (5.2%) were reinitiated with NAs. The median ALT and HBV‐DNA level of patients who needs to reinitiate treatment were 163 U/L and 2 × 10^5^ IU/mL respectively. Biochemical normalisation of patients with the respective follow‐up data occurred until a median of 8 weeks.

**FIGURE 1:**
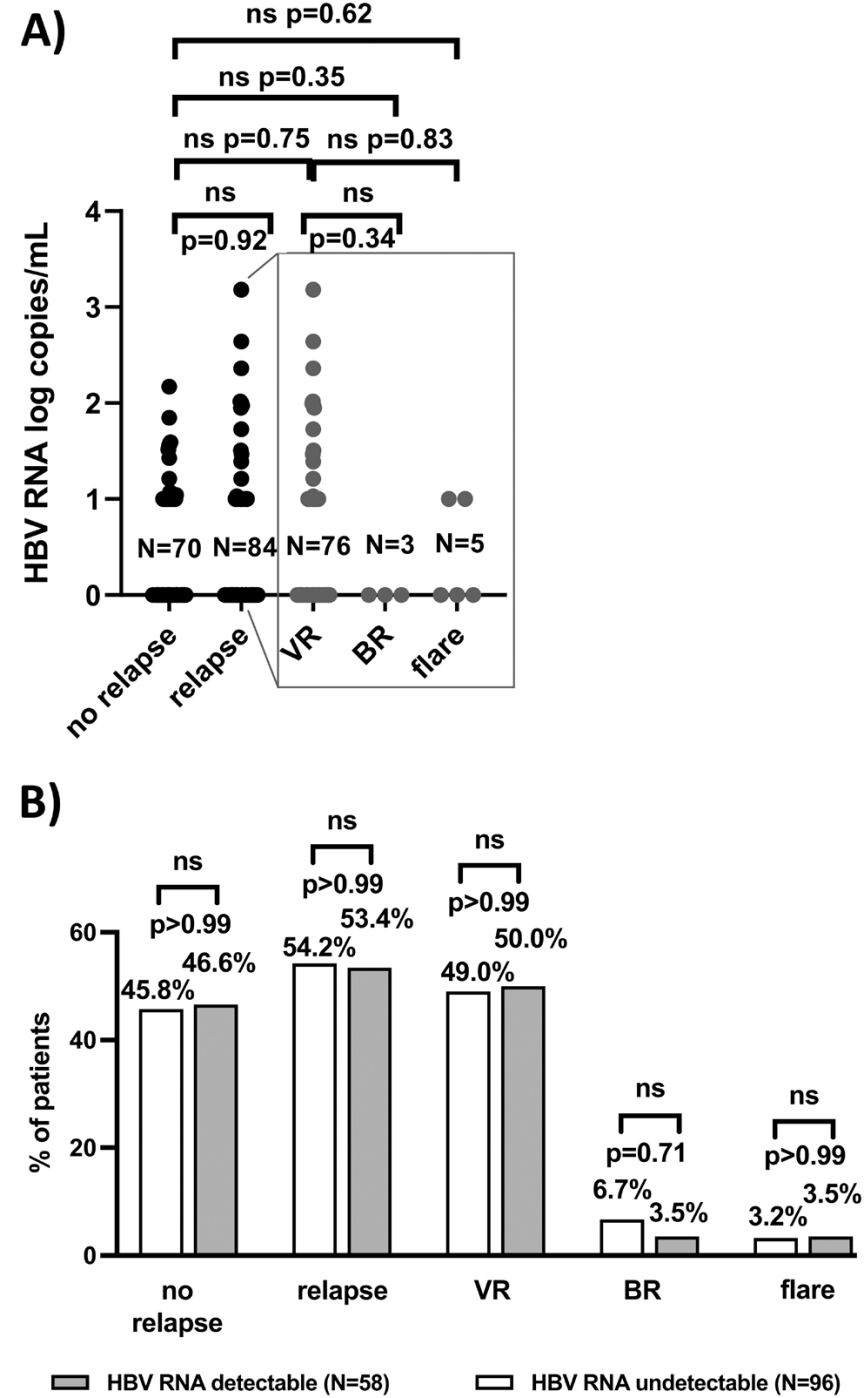
(A) Differences in HBV‐RNA baseline levels between off‐treatment responders and relapsers and (B) proportion of patients without and with relapse 24 weeks after EoT. BR = biochemical relapse; ns = not significant; VR = virological relapse.

HBV‐RNA BL level did not differ significantly between relapsers (median = 0 copies/mL [0–1520 copies/mL]) and off‐treatment responders (median = 0 copies/mL [0–149 copies/mL]) (*p* = 0.92). Subanalyses revealed no significant differences in BL HBV‐RNA levels between off‐treatment responders and patients with VR (median = 0 copies/mL [0–1.5 × 10^3^ copies/mL]) (*p* = 0.75), BR (median = 0 copies/mL [0–0 copies/mL]) (*p* = 0.35) or flare (median = 0 copies/mL [0–10 copies/mL]) (*p* = 0.62) respectively. Additionally, no significant difference in BL HBV‐RNA levels between participants with VR and either BR (*p* = 0.34) or flare (*p* = 0.83) was observed (*p* = 0.37) (Figure 1A).

The proportion of participants with detectable and undetectable HBV‐RNA at BL did not differ significantly within the group of patients with (i) off‐treatment response (detectable HBV‐RNA *N* = 27/58, 46.6%; undetectable HBV‐RNA *N* = 44/96, 45.8%; *p* > 0.99), (ii) relapse (detectable HBV‐RNA *N* = 31/58, 53.4%; undetectable HBV‐RNA *N* = 52/96, 54.2%; *p* > 0.99), (iii) VR (detectable HBV‐RNA *N* = 29/58, 50.0%; undetectable HBV‐RNA *N* = 47/96, 49.0%; *p* > 0.99), (iv) BR (detectable HBV‐RNA *N* = 2/58, 3.5%; undetectable HBV‐RNA *N* = 6/96, 6.7%; *p* = 0.71) or (v) flare (detectable HBV‐RNA *N* = 2/58, 3.5%; undetectable HBV‐RNA *N* = 3/96, 3.2%; *p* > 0.99) respectively (Figure 1B). Considering the small number of patients with BR and flare, these endpoints were treated together with VR as ‘relapse’ in the further analysis.

Importantly, vaccination in the ABX 203‐002 cohort showed no impact on relapse (*p* = 0.71). Furthermore, no significant difference was observed between HBV‐RNA levels before vaccination (median = 0 copies/mL [10–1.5 × 10^3^ copies/mL]) and at EoT (median = 0 copies/mL [10–2.37 × 10^3^ copies/mL]) in the vaccinated participants of the ABX 203‐002 study (*p* = 0.38). Another subanalyses of exclusively nonvaccinated patients revealed no significant difference in BL levels of HBV‐RNA (relapsers: median = 0 copies/mL [0–94.4 copies/mL]) and off‐treatment responders (median = 0 copies/mL [0–149 copies/mL]) (*p* = 0.96) or in the rate of detectable HBV‐RNA to BL in patients with relapse (detectable HBV‐RNA *N* = 13/38, 34.2%; undetectable HBV‐RNA *N* = 25/38, 65.8%) or off‐treatment response (detectable HBV‐RNA *N* = 8/23, 34.8%; undetectable HBV‐RNA *N* = 15/23, 65.2%) (*p* > 0.99).

### 3.3 HBV‐RNA Level and the Prediction of Relapse Dependent on NUC Therapy

Since the timing of relapse differs between patients receiving ETV versus TDF treatment, subanalyses were performed for patients with the respective treatment.

Indeed, out of 60 patients receiving TDF, 80% (*N* = 48) showed a relapse within 24 weeks after EoT while in ETV‐treated patients, the relapse rate was significantly lower (37%, *N* = 35, *p* < 0.0001). Furthermore, a significant difference was found in the rate of detectable HBV‐RNA at BL between patients treated with either ETV (*N* = 43/94, 45.7%) or TDF (*N* = 17/60, 28.3%, *p* = 0.0417). However, no significant difference in the cumulative relapse rate dependent on the HBV‐RNA detectability in patients treated with ETV was observed (relapse + HBV‐RNA detectable: *N* = 17/35, 48.6%, relapse + HBV‐RNA undetectable: *N* = 18/35, 51.4%; *p* > 0.99). In patients treated with TDF, the rate of relapse was even significantly higher in patients with undetectable HBV‐RNA levels at BL (relapse + HBV‐RNA detectable: *N* = 14/48, 29.2%, relapse + HBV‐RNA undetectable: *N* = 34/48, 70.8%; *p* < 0.0001).

### 3.4 HBV‐RNA Level and the Prediction of Relapse Dependent on the HBV Genotype

**FIGURE 2:**
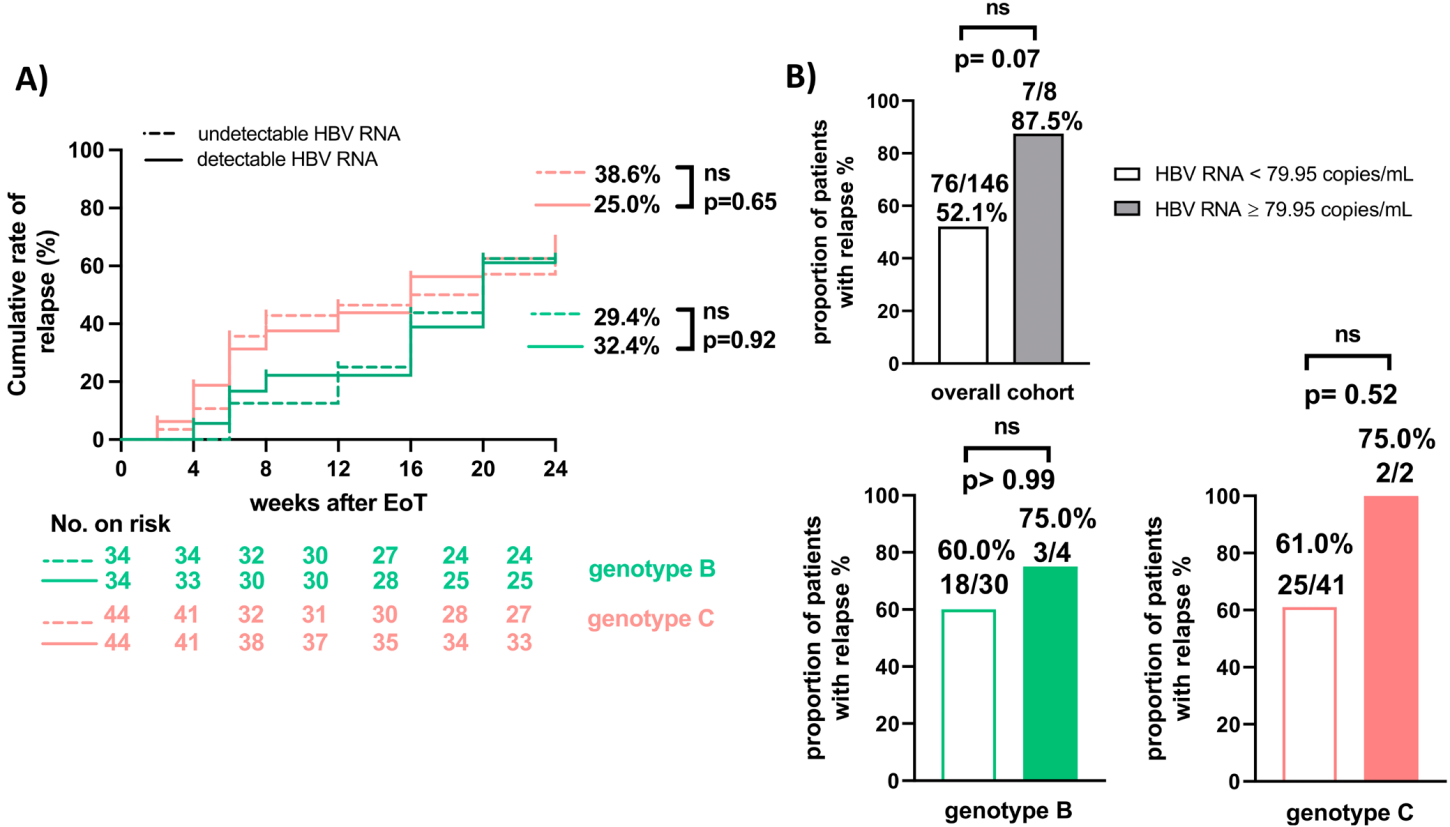
(A) cumulative relapse rates of patients infected with genotype B or C based on HBV‐RNA detectability at BL and (B) proportions of patients with relapse according to the optimal HBV‐RNA cut‐off of 79.95 copies/mL in the overall cohort as well as in subpopulations infected with genotype B or C respectively. Ns = not significant.

No significant differences were found in the rate of detectable HBV‐RNA at BL between patients infected with the respective genotypes (B: *N* = 18/44, 40.9%; C: *N* = 16/34, 47.1%, *p* = 0.17) or in the relapse rates between participants infected with genotype B (*N* = 21/34, 61.8%) or genotype C (*N* = 28/44, 63.6%) (*p* > 0.99) respectively. Furthermore, no significant differences were observed by comparing the cumulative relapse rates dependent on the HBV‐RNA detectability between patients infected with either genotype B (relapse + HBV‐RNA detectable: *N* = 11/34, 32.4%, relapse+ HBV‐RNA undetectable: *N* = 10/34, 29.4%; *p* = 0.92) or C (relapse + HBV‐RNA detectable: *N* = 11/44, 25.0%, relapse + HBV‐RNA undetectable: *N* = 17/44, 38.6%; *p* = 0.65) (Figure 2A).

Using the Youden index, the optimal HBV‐RNA cut‐off to predict relapse was 79.95 copies/mL (sensitivity 98.6%, specificity 8.4%) in the presented cohort. However, the performed ROC analyses revealed an AUC of only 0.5039, *p* = 0.93 (Figure S2A). Using the optimal HBV‐RNA cut‐off, the proportion of study participants with relapse differed not significantly between patients with HBV‐RNA levels < 79.95 copies/mL (*N* = 76/146, 52.1%) or ≥ 79.95 copies/mL (*N* = 7/8, 87.5%) (*p* = 0.07). Similar results were obtained when subanalyses with an HBV‐RNA BL cut‐off of 79.95 copies/mL in the subcohorts of patients infected with genotypes B (< 79.95 copies/mL: *N* = 18/30, 60.0%; ≥ 79.95 copies/mL: *N* = 3/4, 75.0%; *p* > 0.99) and C (< 79.95 copies/mL: *N* = 25/41, 61.0%; ≥ 79.95 copies/mL: *N* = 2/2, 100.0%; *p* = 0.52) were performed (Figure 2B).

Patients infected with either genotype A (*N* = 3, 1.9%), D (*N* = 11, 7.1%) or E (*N* = 1, 0.6%) were excluded from the analyses due to the low patient numbers. Of note, of 11 subjects infected with genotype D, nine patients sustained relapse (81.8%), but all nine patients had HBV‐RNA level < 10 copies/mL at BL (in 77.8%, HBV‐RNA was undetectable and 22.2% had HBV‐RNA level < 10 copies/mL). In addition, out of five patients with known genotype and clinical relapse, four (80%) were infected with genotype D.

### 3.5 Combined HBV Biomarkers and the Prediction of Relapse

Finally, we investigated if the significance of HBV‐RNA in the prediction of relapse could be improved by combining HBV‐RNA levels with predefined HBV biomarker cut‐offs (HBcrAg: ≤/> log 2 IU/mL, HBsAg: ≤/> 100 IU/mL) [20, 21]. For the ABX 203‐002 cohort, an anti‐HBc cut‐off of ≥ 325 IU/mL was described before as the optimal cut‐off value for the prediction of relapse [27]. Using the Youden index, this cut‐off was again identified as the optimal cut‐off to predict relapse when the ABX 203‐002 and Terminator 2 cohort were combined for the presented analyses (anti‐HBc level was determined in 149/154 patients, 96.8%, ROC analyses: sensitivity 51%, specificity 71%, AUC = 0.6039, *p* = 0.0287, Figure S2B). The proportion of patients categorised according to the predefined HBsAg cut‐off value of 100 IU/mL and HBcrAg cut‐off value of 2 log U/mL or according to the determined optimal anti‐HBc cut‐off value of 325 IU/mL is presented in Table S2.

**FIGURE 3:**
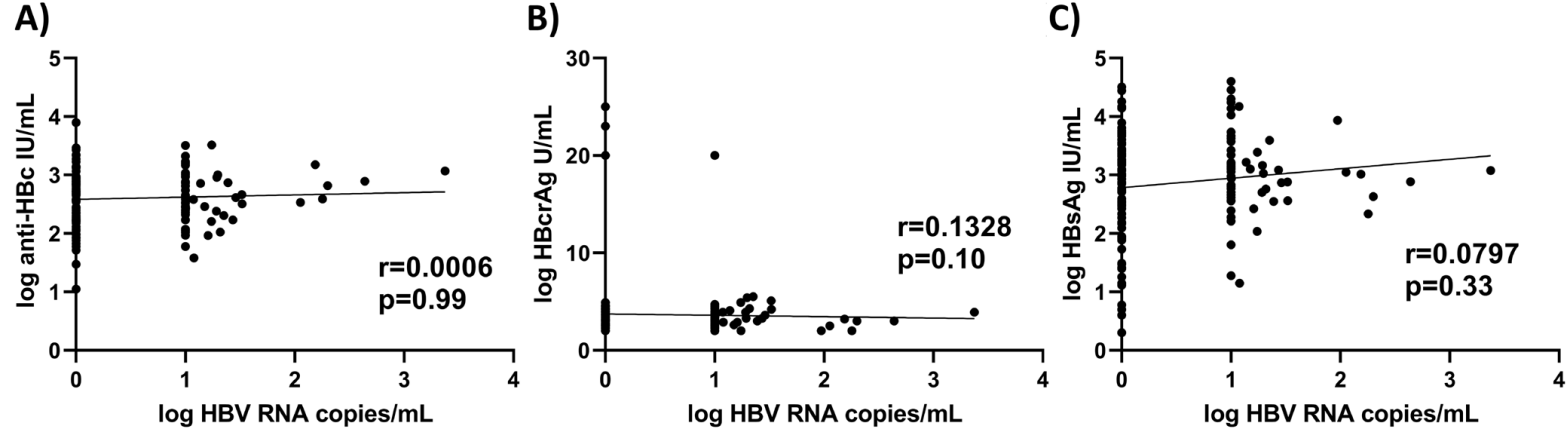
Correlation analyses between BL HBV‐RNA levels with BL levels of (A) anti‐HBc, (B) HBcrAg and (C) HBsAg.

Correlation analyses did not reveal significant correlations between HBV‐RNA BL levels and anti‐HBc BL levels (*r* = 0.0006, *p* = 0.99), HBcrAg BL levels (*r* = 0.1328, *p* = 0.10) or HBsAg BL levels (*r* = 0.0797, *p* = 0.33) respectively (Figure 3A–C).

By analysing the proportions of study participants with and without relapse according to the investigated biomarker cut‐offs significant differences were observed. Relapse rates were significantly lower in patients with HBsAg level of ≤ 100 IU/mL (*N* = 4/16, 20%) at BL compared to patients with an HBsAg level of > 100 IU/mL (*N* = 79/132, 59.9%) (*p* = 0.0013). Regarding BL anti‐HBc levels, relapse occurred in 39.0% (*N* = 23/59) of patients with an anti‐HBc level < 325 IU/mL, while this was the case in 61.1% (*N* = 55/90) of those with values ≥ 325 IU/mL (*p* = 0.0117). Of note, 80% (*N* = 4/5) of patients with flare (80%) had anti‐HBc level < 325 IU/mL.

In contrast, proportions of participants who relapsed did not differ significantly between the group of patients with HBcrAg BL levels ≤ 2 log U/mL (*N* = 12/25, 48%) and > 2 log U/mL (*N* = 70/128, 54.7%) (*p* = 0.66) respectively.

**FIGURE 4:**
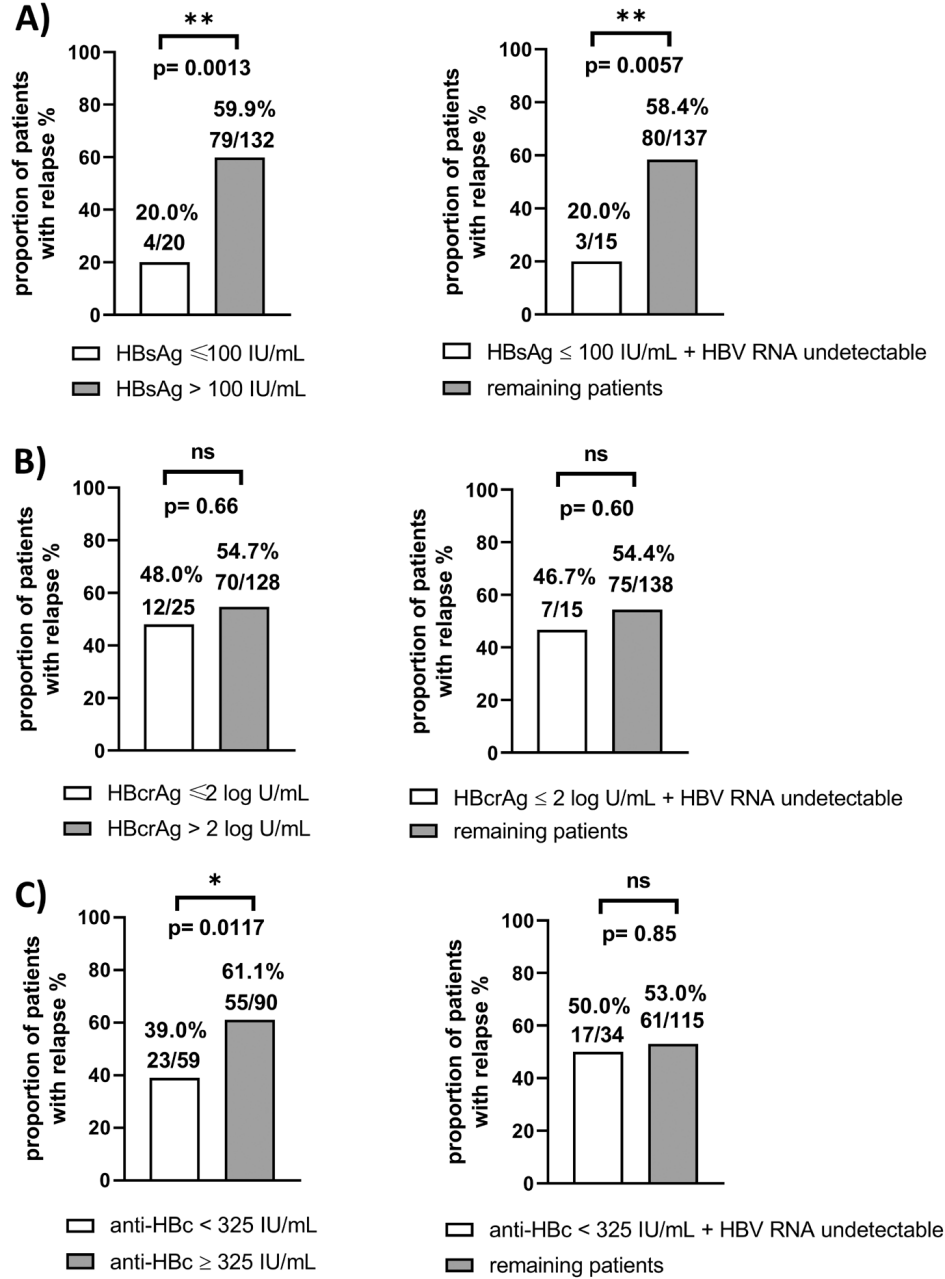
Proportion of patients with relapse 24 weeks after EoT according to BL levels of selected HBV biomarkers alone and in combination with HBV‐RNA BL levels (A) HBsAg, (B) HBcrAg and (C) anti‐HBc. Ns = not significant.

Importantly, combining undetectable HBV‐RNA levels with other HBV biomarker cut‐offs (HBsAg 100 IU/mL, HBcrAg 2 log U/mL, anti‐HBc 325 IU/mL) did not improve relapse prediction. In detail, combining undetectable HBV‐RNA BL levels with HBsAg ≤ 100 IU/mL, three of 15 patients (20%) relapsed until week 24 after EoT, while 58.4% (*N* = 80/127) with biomarkers under the respective levels did not (*p* = 0.0057). Combining HBcrAg ≤ 2 log U/mL and undetectable HBV‐RNA BL levels resulted in invariable not significant differences in the proportions of participants with relapse (HBcrAg ≤ 2 log U/mL + undetectable HBV‐RNA = 46.7%, *N* = 7/15; remaining patients = 54.4%, 75/138; *p* = 0.60). Regarding the combination of undetectable HBV‐RNA with anti‐HBc < 325 IU/mL, the differences in the proportions of patients with relapse (anti‐HBc < 325 IU/mL + undetectable HBV‐RNA = 50%, *N* = 17/34; remaining patients = 53%, *N* = 61/115) lost significance (*p* = 0.85) (Figure 4A–C). Furthermore, the combination of three or even all four of the biomarkers did not reveal significant differences in proportions of patients with relapse (Figure S3A–E). Of note, the combination of anti‐HBc and HBsAg resulted in a significantly different proportion of patients with relapse (anti‐HBc < 325 IU/mL + HBsAg = 12.5%, *N* = 1/8; remaining patients = 54.6%, *N* = 77/141, *p* = 0.0260), while the comparison to the proportions of patients with relapse with HBsAg levels ≤ 100 IU/mL alone showed no significant results (HBsAg = 20.0%, *N* = 4/20; *p* = 0.41). However, regarding the low number of patients with the respective low biomarker levels, this result needs to be further investigated (Figure S3F,G).

### 3.6 Binary Logistic Regression Analyses of Selected Risk Factors for the Prediction of Relapse

Consistent with the results of the performed analyses, multivariate regression analysis revealed only low HBsAg BL level (HR = 2.057 with CI = 1.306–3.240, *p* = 0.002) and a low anti‐HBc BL level (HR = 2.230 with CI = 1.063–4.680, *p* = 0.034) as independent predictors for relapse after therapy stop (Table 2).

**TABLE 2:** | Binary logistic regression analysis of risk factors for the prediction of relapse.

	Relapse (*n* = 83)					
	Univariate			Multivariate		
	HR	95% CI	*p*	HR	95% CI	*p*
Age (year)	0.980	0.946–1.015	0.259			
Treatment duration (month)	1.000	0.992–1.009	0.921			
Genotype						
B	0.641	0.293–1.400	0.264			
C	0.550	0.267–1.134	0.105			
D	0.232	0.048–1.113	0.068			
HBV‐RNA (log cp/mL)	1.586	0.963–2.612	0.070			
HBV‐RNA undetectable	1.554	0.810–2.979	0.184			
HBV‐RNA < 79.95 (cp/ml)	5.455	0.641–46.435	0.121			
Anti‐HBc (log IU/mL)	2.345	1.164–4.724	0.017	2.230	1.063–4.680	0.034
HBsAg (log IU/mL)	2.131	1.363–3.331	< 0.001	2.057	1.306–3.240	0.002
HBcrAg (log U/mL)	1.095	0.950–1.262	0.212			

## 4 Discussion

Nucleos(t)ide analogues achieve a virological and biochemical response in most chronically infected HBV patients. Especially for HBeAg‐negative cHBV patients, international guidelines consistently recommend the permanent treatment with NA until HBsAg loss, but functional cure is only a rare event under NA treatment [8, 9]. Therefore, other HBV biomarkers that help to estimate a safe NA withdrawal before HBsAg seroclearance are of high clinical interest.

The performed retrospective analysis of an exclusively HBeAg‐negative patient cohort, obtained from a multicentre, prospective study as well as a prospective observational study, investigated the HBV‐RNA level at EoT as a marker for the prediction of relapse after NA cessation.

Low or undetectable HBV‐RNA levels at EoT (alone or in combination with either HBsAg or HBcrAg levels) were associated with a lower risk of VR or BR after NA cessation in cohorts of HBeAg‐positive patients or mixed cohorts of HBeAg positive and negative patients [17, 18, 19, 30, 31]. So far, only four studies with small cohorts of exclusively HBeAg‐negative patients were performed in the context of relapse prediction by the HBV‐RNA level at the end of NA treatment [20, 21, 22, 23]. All studies reported an association between detectable HBV‐RNA levels at EoT and a higher risk of BR after treatment cessation. In contrast, the results concerning a higher risk of VR after treatment cessation remained discrepant. Laras et al. [22], investigating HBeAg‐negative patients exclusively infected with genotype D, reported the association between undetectable HBV‐RNA levels at EoT and a lower risk for VR and BR 12 months after treatment cessation [22]. In contrast, Papatheodoridi et al. [21] found an association between detectable HBV‐RNA levels at EoT and BR 12 months after therapy stop, but VR could only be significantly associated with HBV‐RNA levels 1 month after treatment cessation. Although the HBV genotype was not determined in this study, the authors stated a supposed genotype D in the majority of patients, due to the Greek patient cohort. In a third study conducted by Carey et al. [23], detectable HBV‐RNA at EoT was associated with severe ALT flares in 4 of 23 patients. (100% specificity, 75% sensitivity). The authors reported no significant influence of the genotype on ALT flares but described a higher frequency (75%) of precore (G1896A) and basal core promotor (A1762T/G1764A) mutations in patients with severe ALT flares after NA cessation. The most recently published study from Thompson et al. [20], investigating 65 HBeAg‐negative patients mainly infected with genotype B or C, reported a significant association between a detectable HBV‐RNA level at EoT and the likelihood of a higher risk of BR and flares 96 weeks after NA withdrawal. In contrast, the association with higher risks of VR remained only significant when an undetectable HBV‐RNA level occurred in combination with HBsAg levels ≤ 100 IU/mL at EoT.

In the presented analyses with the so far largest cohort of HBeAg‐negative patients, we could confirm that HBV‐RNA levels at EoT are not significantly associated with the risk of VR. But interestingly, we did not find an association to the risk of BR or flares 24 weeks after therapy cessation. Furthermore, the prediction of relapse events could not be improved by the combination of HBV‐RNA with other HBV biomarkers, namely, HBsAg, HBcrAg and anti‐HBc.

Several factors, explaining the discrepant results to other studies seem to be reasonable. First, the proportion of patients with BR or flare was smaller in our cohort compared to the above‐mentioned cohorts in follow‐up periods of 52–96 weeks after EoT. Although the shorter follow‐up period of 24 weeks in the presented study might underestimate the proportions of patients with VR and particularly BR, most patients experience relapse in the first 6 months after therapy withdrawal [8]. Of note, this was confirmed also in the subcohort of participants of the Terminator 2 cohort with a follow‐up period of up to 130 weeks after EoT at the timepoint of analysis, where no further case of relapse occurred after 24 weeks of NA cessation. Therefore, the investigated follow‐up period of 24 weeks is representative for the majority of cases and further factors might contribute to the higher rates of BR reported from the respective studies.

One remarkable difference in the BL characteristics of the respective HBeAg‐negative cohorts is the low proportion of patients infected with genotype D in the presented study. Although strong evidence is missing, some studies reported a significant role of the HBV genotype in the course of liver disease. Wai et al. reported an independent association of genotype D with the development of hepatitis B–related acute liver failure in 34 patients [32] and in a study from India genotype D was related to more advanced liver disease compared to other genotypes [33]. Although not investigated so far, a correlation between the genotype and the rates of BR after NA cessation seems to be reasonable. Importantly, out of four patients with known genotype and clinical relapse, 80% were infected with genotype D in our study. However, as a limitation of the presented study, genotypes A, D and E were underrepresented, and similar proportions of patients infected with the reported genotypes are necessary to support the above‐mentioned hypothesis of a genotype‐dependent significance for HBV‐RNA levels in the prediction of relapse after NA cessation.

Variants of HBV with precore stop codon mutations are usually associated with HBeAg‐negative cHBV [34]. From some studies also evidence is raising that additional mutations in the core region, frequently accompanying precore mutations, may enhance HBV virulence by building a major target of cytotoxic T lymphocytes [35, 36, 37]. Therefore, it would have been interesting to also identify the precore and core regions of cases with and without relapse in our cohort and further studies are necessary to identify differences in the mutation profile of different viral genotypes. But obviously, next to the viral aspects that may have an impact on the outcome relapse after NA cessation, the immunological factors of the hosts need to be considered in answering this complex question.

One may argue that the therapeutic vaccination that 60.1% of patients received may limit the relapse rates in our cohort as well as the comparability to the general population. Although already discussed elsewhere [27], it is noteworthy to mention again that HBV‐RNA levels were measured before vaccination. Therefore, the design of the presented study excluded the impact of the vaccination on the biomarker level. Furthermore, relapse rates did not differ significantly between the vaccinated group (*N* = 43/91, 47%) and the nonvaccinated group (*N* = 23/43, 53%) of the ABX 203‐002 cohort (*p* = 0.71).

Finally, some limitations in the study design have to be mentioned. The study results were based on a single HBV‐RNA value at baseline and the timepoints of HBV‐RNA measurement differed between the two cohorts. Furthermore, quantification of HBV‐RNA was performed by using the investigational Cobas 6800/8800 HBV‐RNA assay. Since the sequence of the primers is not published, the quantified form of HBV pgRNA (3′‐truncated vs. 3.5‐kb HBV‐RNA) cannot be specified, which may limit the comparability to other studies.

However, to our knowledge, the presented study is the one with the largest cohort of exclusively HBeAg‐negative patients investigating the impact of HBV‐RNA levels at EoT as a predictor of relapse after NA cessation. Although the rate of patients with BR is low in our study, the rate of HBV‐RNA detectability (37.7%, *N* = 58/154) is significantly higher compared to the previously performed studies. Therefore, the results of the missing association between VR and detectable HBV‐RNA at the timepoint of therapy withdrawal seem to be reliable. Furthermore, of eight patients with BR, including five patients with flare, six patients (75%) had undetectable HBV‐RNA levels at EoT and two patients (25%) had HBV‐RNA levels under the lower limit of quantification (≤ 10 copies/mL), representing the importance of the further evaluation of the predictive value of EoT serum HBV‐RNA levels in the prediction of relapse. Furthermore, the presented study is the first one performing subanalyses with appropriate numbers of HBeAg‐negative patients, infected with either genotype B or C. At least, even without significant results, the combination of anti‐HBc levels in combination with HBV‐RNA levels as a reliable predictor for relapse was investigated.

In conclusion, in HBeAg‐negative patients, infected mainly with HBV genotypes B and C, HBV‐RNA levels at EoT were not valuable for the prediction of VR or BR 24 weeks after NA cessation. The results contrast with data from some previously studied HBeAg‐negative cohorts. The heterogeneity of the results indicates the necessity of further confirmation of the predictive value of EoT serum HBV‐RNA levels in the prediction of relapse.

## Ethics Statement

The ABX 203‐002 study was approved by international ethics committees (ABX‐203, NCT02249988). The presented analysis of biomarker and anonymous patient data was additionally approved by the local ethics committee of Hannover Medical School (10556_BO_K_2022). Likewise, the observational register trial Terminator 2 was approved by the local ethics committee of Hannover Medical School (7982_BO_K_2018).

## Consent

All subjects that were included in the ABX 203‐002 study or in the Terminator 2 trial gave written informed consent for study participation. Only data of patients who participated in the additional serum sampling with sufficient clinical data and serum samples were included in the presented study.

## Conflicts of Interest

The authors declare no conflicts of interest.

## Supporting information


Appendix S1.

## Data Availability

The data that support the findings of this study are available from the corresponding author upon reasonable request.
