# Supplementary material for: Limited Value of HBV‐RNA for Relapse Prediction After Nucleos(t)ide Analogue Withdrawal in HBeAg‐negative Hepatitis B Patients
Source: J Viral Hepat. 2024 Oct 19;32(4):e14026. doi: 10.1111/jvh.14026 (PMC11887418; doi:10.1111/jvh.14026)
Supplement: Supplementary file 1 — Appendix S1. [file JVH-32-0-s001.docx]

**Supplement**

**Suppl. table 1** Baseline characteristics of the respective sub-populations.

| Parameter |  | N (total cohort=154) | |
| --- | --- | --- | --- |
|  |  | ABX 203-002 (N=136) | Terminator 2 (N=18) |
| Age (in years) |  | 53 (20-66) | 50 (39-63) |
| Male sex |  | 102 (75 %) | 13 (72.2 %) |
| Nucleos(t)id analogue | Entecavir  Tenofovir | 90 (66.2 %)  46 (33.8 %) | 4 (22.2 %)  14 (77.8 %) |
| Therapy duration (in month) |  | 56 (30-190) | 101.5 (42-257) |
| HBeAg negativity |  | 136 (100 %) | 18 (100 %) |
| HBV DNA (<40 IU/mL) |  | 136 (100 %) | 18 (100 %) |
| ALT (U/mL) |  | 20.5 (6-55) | 21.5 (6-71) |
| Transient elastography (kPa) |  | 5.2 (3.1-9.9) | 5.2 (2.7-9.9) |
| Platelets (thousand/µl) |  | 194 (81-332) | 219.5 (138-348) |
| Bilirubin (mmol/l) |  | 8 (3-19) | 9.5 (3-36) |
| Anti-HBc level (IU/mL) |  | 445 (11.2-7.9x10^3^) | 280.5 (56-4.2x10^3^) |
| HBsAg level (IU/mL) |  | 801 (2-3.2x10^4^) | 2.2x10^3^ (124-4.0x10^4^) |
| HBcrAg level (log U/mL) |  | 3.2 (2.0-5.5) | 2.0 (2.0-4.6) |
| HBV RNA (copies/mL) |  | 0 (0-2.4x10^3^) | 0 (0-94.4) |
| **Genotype** |  |  |  |
| A |  | 2 (1.5%) | 1 (5.6%) |
| B |  | 34 (25.0 %) | 0 (0 %) |
| C |  | 43 (31.64%) | 1 (5.6%) |
| D |  | 0 (0%) | 11 (61.1%) |
| E |  | 0 (0%) | 1 (5.6%) |
| unknown |  | 54 (39.7%) | 4 (22.2%) |

**Suppl. Table 2** Proportion of patients categorized according to the pre-defined biomarker cut-off values.

| HBV biomarker | N (total cohort=154) (%) |
| --- | --- |
| anti-HBc level (IU/mL) | (N=149) |
| < 325 IU/mL | 59 (39.6%) |
| ≥ 325 IU/mL | 90 (60.4%) |
| HBsAg level (IU/mL) | (N=152) |
| ≤ 100 IU/mL | 20 (13.2%) |
| > 100 IU/mL | 132 (86.8%) |
| HBcrAg level (log U/mL) | (N=153) |
| ≤ 2 log U/mL | 25 (16.3%) |
| > 2 log U/mL | 128 (83.7%) |
| HBV RNA (copies/mL) | (N= 154) |
| undetectable | 96 (62.3%) |
| detectable | 58 (37.7%) |

**
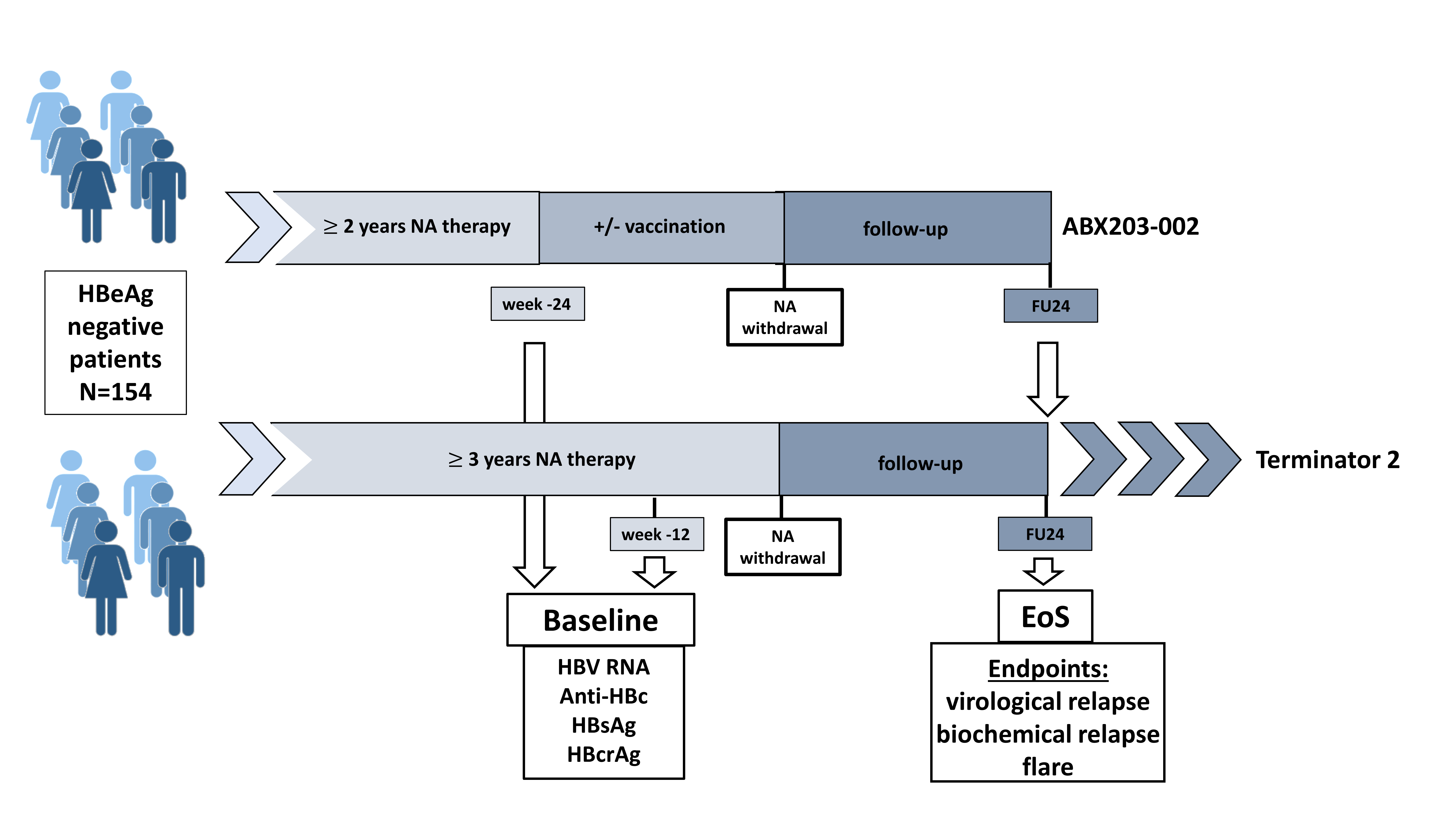
**

**Suppl. figure 1** Overview of the design of the presented study. Data and blood samples of HBeAg negative patients participating either in the ABV203-002 study or in the Terminator 2 register trial were analyzed in the analysis. The figure is adapted from Fig. 1 published by Wübbolding et al. ^21^.

**
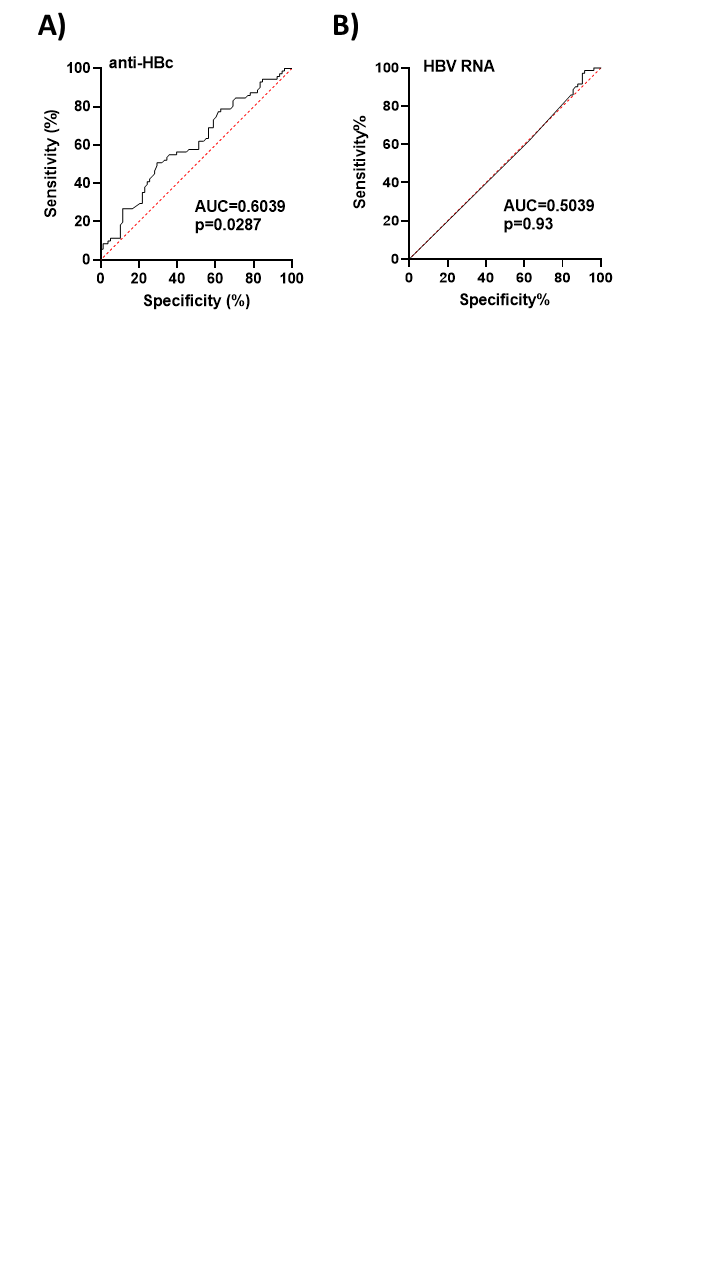
**

**Suppl. figure 2** Area under the receiver operating characteristic (AUROC) curves of end of treatment A) anti-HBc and B) HBV RNA for the prediction of relapse after NA cessation.


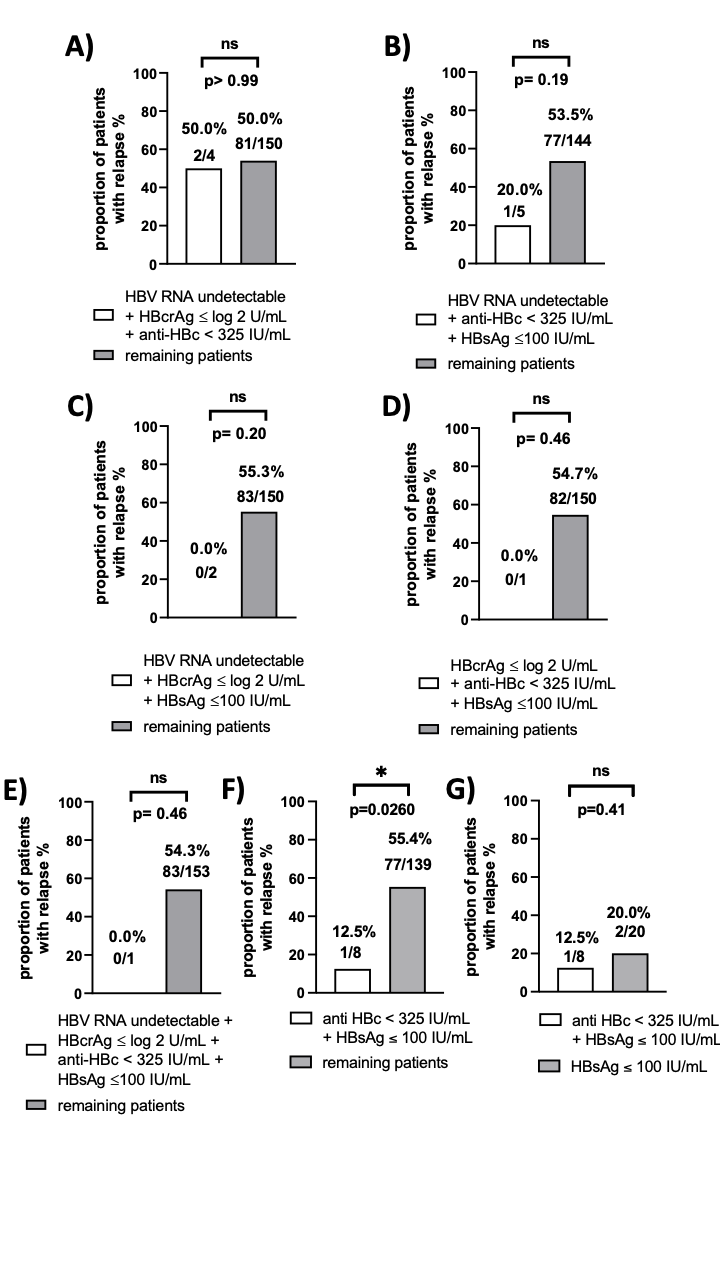


**Suppl. figure 3** Proportion of patients with relapse 24 weeks after end of treatment according to the predefined BL levels of A) HBV RNA + HBcrAg + anti-HBc, B) HBV RNA + anti-HBc + HBsAg, C) HBV RNA + HBcrAg+ HBsAg, D) HBcrAg + anti-HBc + HBsAg, E) HBV RNA + HBcrAg + anti-HBc + HBsAg, F) anti-HBc and HBsAg and G) anti-HBc + HBsAg vs. HBsAg alone.
